# Molecular identification and mitogenome character of illegally traded tufted deer, *Elaphodus cephalophus cephalophus*

**DOI:** 10.1080/23802359.2020.1821817

**Published:** 2020-09-16

**Authors:** Ning Han, Luxin Zhang, Xuhui Wei, Jing Liu

**Affiliations:** Jincheng College of Sichuan University, Chengdu, China

**Keywords:** Complete mitogenome, *Elaphodus cephalophus cephalophus*, phylogenetic analysis

## Abstract

The total mitogenome of the confiscated material of tufted deer, *Elaphodus cephalophus cephalophus* was sequenced and annotated. It is 16,351bp in length and contained 2 ribosomal RNAs, 22 transfer RNAs (tRNAs), 13 protein-coding genes (PCGs) and, 1 control region (CR). Phylogenetic analyses showed the close relationship of *Elaphodus* and *Muntiacus*, and monophyletic clades of genera *Elaphodus* and *Muntiacus*.

*Elaphodus cephalophus* is a unique species of genus *Elaphodus* (monotypic) from a large area of southern China and northwestern Myanmar (Leslie et al. 2013) with three subspecies, *E. c. cephalophus, E. c. ichangensis* and *E. c. michianus* (Wang 2003). However, there are still controversies on the distribution of these subspecies. Among these three subspecies, *E. c. cephalophus* is distributed in western China (Wang 2003; Wang et al. 2007). Due to the overhunting and trading in remote areas, the species has been listed as ‘Near Threatened’’ by the IUCN (Harris and Jiang 2015). Despite available mitogenome of tufted deer (Pang et al. 2008; Zhang et al. 2019), reference data for the three subspecies mitogenome are still lacking at present. In this study, we determined and characterized the complete mitogenome of *E. c. cephalophus* and investigate the phylogenetics of the species with published homologous mitogenome genes of other related species.

The illegally traded tufted deer with only broken leg parts was confiscated material by local public security bureau for forest. The specimen studied here was collected from Dujiangyan (N30°59, E103°33), Sichuan Province, China, in 2019, which was deposited in the Museum of public security bureau of forest in Dujiangyan (Specimen voucher: DJY2019006). Species molecular identification was done by reference sequences of *Elaphodus cephalophus* retrieved from GenBank (Wang and Lan 2000; Pang et al. 2008). Subspecies affiliation (*E. c. cephalophus*) was identified according to geographic locality (poaching site) provided by local public security bureau of forest, in combination with known subspecies distribution of the three tufted deer subspecies (Wang 2003; Pan et al. 2007; Wang et al. 2007). The leg muscle tissue was preserved in absolute ethanol and stored in −80 °C refrigerator. DNA from muscle tissue was isolated using the Ezup pillar genomic DNA extraction kit (Sangon Biotech, Shanghai, China). The mitogenome was amplified in 11 overlapping segments by PCR with 11 sets of primers designed by Pang et al (Pang et al. 2008).

The total mitogenome sequence of *E. c. cephalophus* (16,351 bp, GenBank accession number: MT726046) contains 13 protein coding genes (PCGs), 2 ribosomal RNA (rRNA) genes, 22 tRNA genes, and a control region (D-loop). The total nucleotide composition of the *E. c. cephalophus* mitogenome is A (33.3%), G (13.4%), C (23.9%) and T (29.4%). Most genes are encoded on heavy strand (H strand), except the *nad6* and eight *tRNAs* (*tRNA-P, Q, A, N, C, Y, S*_2_, and *E*) are located on the L-light strand (L strand). Nine of the protein coding genes (PCGs) began with ATG, except *nad2*, nad3, and *nad5* with ATT, and *nad4l* with GTG. Three PCGs (*nad2, atp8* and *cytb*) ended with TAG, and six (*nad1*, *cox1, cox2, atp6*, *nad4l, nad5* and *nad6*) terminated by TAA. For the incomplete stop codon, two PCGs (*cox3* and *nad3*) ended with TA, whereas *nad4* stopped with a single base T. 12S and 16S rRNA are 957 and 1566 bp in length, respectively.

Phylogenetic analysis was performed using 11 genera (*Rusa*, *Przewalskium*, *Cervus*, *Rucervus*, *Elaphurus*, *Axis*, *Elaphodus*, *Muntiacus*, *Rangifer*, *Alces*, and *Moschus*) based on combined PCGs. The Maximum likelihood tree was reconstructed by MEGA7.0 (Kumar et al. 2016) with *Moschus berezovskii* as outgroup (Figure 1). The newly sequenced *E. c. cephalophus* (Dujiangyan population) and *E. cephalophus* (DQ873526) from unknown locality formed a well-supported clade that was the sister clade to the Yingjing county population (MN251783). Our results also indicated that the geographic differentiation corresponds to the mitochondrial preliminary differences of these populations. Genus *Elaphodus* was closely related to the *Muntiacus*, and the genera (*Elaphodus* and *Muntiacus*) are monophyletic with high bootstrap support. The mitogenome data of subspecies *E. c. cephalophus* will benefit the three subspecies identification and support future effective conservation and management strategies plans by authorities.

**Figure 1.**
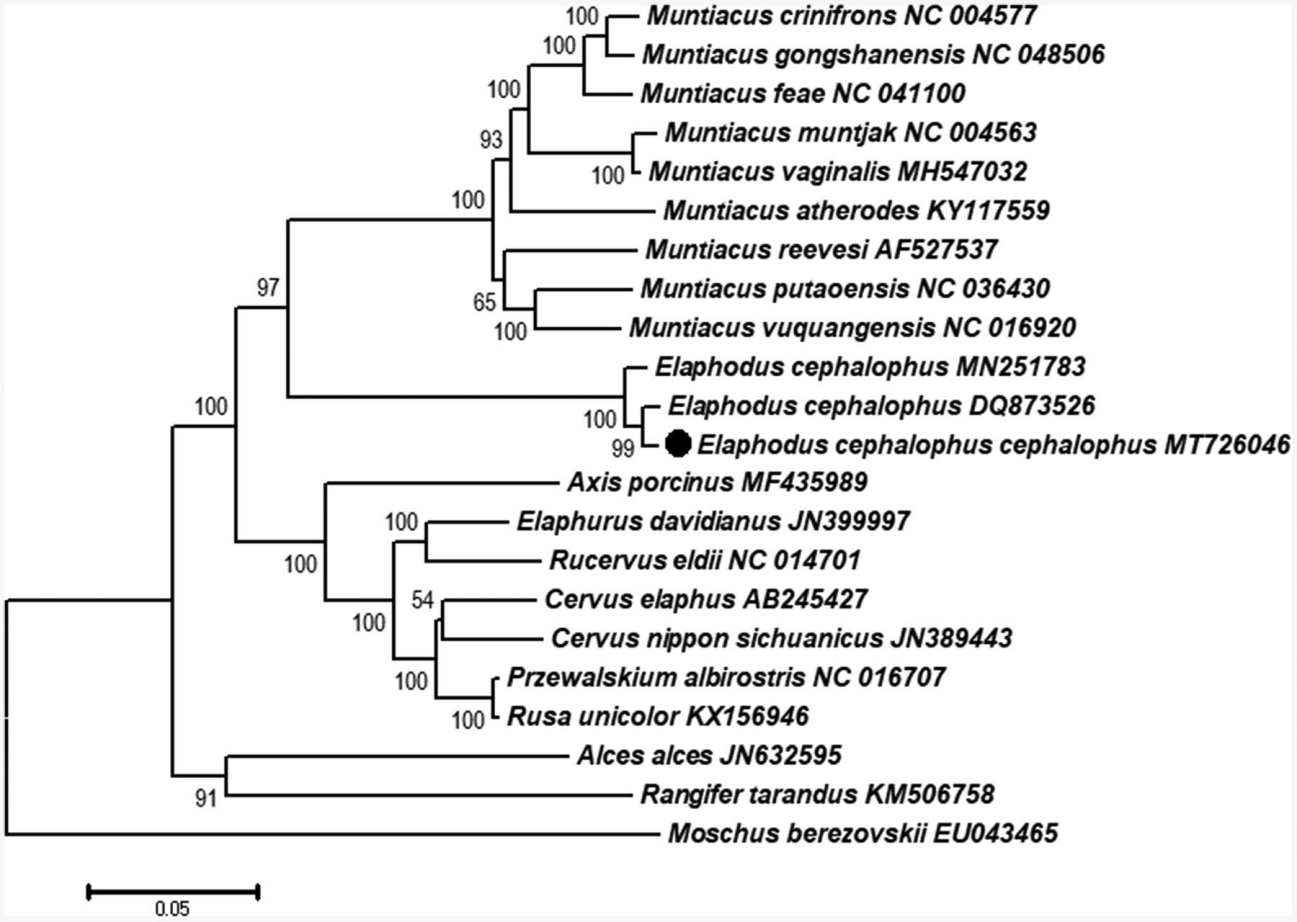
Maximum likelihood tree inferred from the concatenated nucleotide sequences of 13 mt PCGs with *Moschus berezovskii* as outgroup. Bootstrap values for the nodes are denoted.

## Data Availability

The data used to support the findings of this study are available in GenBank at https://www.ncbi.nlm.nih.gov/nuccore/MT726046, reference number MT726046.
